# Comprehensive swallowing exercises to treat complicated dysphagia caused by esophageal replacement with colon: A case report

**DOI:** 10.1097/MD.0000000000005707

**Published:** 2017-02-10

**Authors:** Li Jiang, Yujue Wang, Na Li, Weihong Qiu, Huixiang Wu, Jianshan Huo, Meng Dai, Yong Yu, Guifang Wan, Zulin Dou, Weiping Guo

**Affiliations:** a Department of Rehabilitation; b Department of Rehabilitation, 3rd Affiliated Hospital of Sun Yat-sen University, Guangzhou, China.

**Keywords:** anastomotic stricture, dysphagia, esophageal replacement with colon, rehabilitation

## Abstract

**Introduction:**

Surgical procedures for colonic replacement of the esophagus are most commonly associated with anastomotic stricture which cause dysphagia. In this report, we describe a rare case of a patient who demonstrated dysphagia resulting from an anastomotic stricture following esophageal replacement with the colon. All the treatments to dilate the anastomotic stricture were ineffective. To investigate the new treatment strategy for a case with complicated dysphagia, clinical dysphagia evaluations, functional oral intake scale (FOIS), videofluoroscopic swallowing study as well as high-resolution manometry were used to evaluate the swallowing function of the patient before and after treatments.

**Interventions:**

Comprehensive swallowing exercises included the protective airway maneuver, tongue pressure resistance feedback exercise, Masako Maneuver swallowing exercise, and the effortful swallowing exercise.

**Outcomes:**

Comprehensive swallowing exercises showed good effect in the patient. The FOIS score increased from level 1 to level 7. The videofluoroscopy digital analysis showed that the pharynx constriction rate was 23% and 50%, before and after treatment, respectively. The data from the high-resolution manometry displayed that: the value of the velopharyngeal pressure peak was 82.8 mmHg before treatment and 156.9 mmHg after treatment; the velopharyngeal contraction duration time was 310 milliseconds before treatment and 525 milliseconds after treatment; the value of the hypopharynx pressure peak was 53.7 mmHg before treatment and 103.2 mmHg after treatment; and the hypopharynx contraction duration time was 390 milliseconds before treatment and 1030 milliseconds after treatment. The swallowing visualization illustrated that a bolus could normally pass through the anastomotic stoma, and the bolus leakage was no longer present. The patient was able to eat various consistencies of food independently, and we were able to remove the jejunum nutrient catheter before discharging the patient.

**Conclusion:**

For patients with dysphagia caused by anastomotic stricture following esophageal replacement with colon, the swallowing function can be improved by enhancing the pharyngeal impetus when treatment using esophageal dilation is ineffective.

## 1 Introduction

The colon is a commonly used conduit for the replacement of the esophagus in patients with esophageal strictures or tumors. Surgical procedures for colonic replacement of the esophagus are most commonly associated with anastomotic stricture complications. A long-term follow-up study indicated that 5% of postoperative patients demonstrated anastomotic stricture in varied degrees,^[1]^ characterized by dysphagia, or furthermore, absent oral intake, weight loss, malnutrition, and other relevant disorders.^[2–4]^ Presently, balloon dilatation, bougienage, and esophageal stent implantation are the recognized treatment options for anastomotic stricture.^[5,6]^ In this report, we describe a rare case of a patient who demonstrated dysphagia resulting from an anastomotic stricture following esophageal replacement with the colon. The patient was admitted to our department on April 9th, 2015, and the medical reports for the 26-year old woman indicated that she was treated using repeated dilatation for the stricture using various approaches. However, she did not display a distinct improvement in swallowing function; furthermore, the patient presented an anastomotic fistula dilatation. She was fed through a jejunum nutrient catheter for 7 years prior to admittance to our department. Based on her medical history and the results of the swallowing assessment, the patient underwent the comprehensive swallowing function exercises to enhance the oropharyngeal bolus propulsion as a novel approach to treating dysphagia caused by esophageal replacement with colon. After undergoing the swallowing function rehabilitation for 43 days, the jejunum nutrient catheter was removed and the patient advanced to complete oral feeding. This study was approved by Clinical Trial Ethics Committee of 3rd Affiliated Hospital of Sun Yat-sen University.

## 2 Case report

### 2.1 Medical history

A 26-year-old woman was admitted to our department with dysphagia resulting from an anastomotic stricture following esophageal replacement with the colon. The patient was subjected to operation due to the presence of an esophageal stromal tumor in 2008. Following the operation, she was treated with continued balloon dilatation (14, 18 mm) for 40 days. There was no distinct improvement in the swallowing function by the patient. Six months following the operation, the patient underwent esophageal stent placement. Due to complications of the stent to remain stably positioned in the esophagus, the esophageal stent placement was unsuccessful. To circumvent these issues, the patient underwent an operation designed to repair the anastomostic stricture with paltysma myocutaneous flaps at the end of 2008. Despite the previously mentioned attempts to overcome the impaired swallowing function, the patient oral nutrient intake remained inadequate. The gastroscopy demonstrated that the anastomotic stricture presented with severe scarring. The diameter of the anastomosis was limited to approximately 0.5 cm. Following the gastroscopy, the patient repeatedly underwent endoscopic balloon dilations 7 times, as well as argon knife scar resections for 3 occurrences. Furthermore, the medical history of the patient indicates that during a gastroscopy conducted in 2010, the gastroscope could not pass the anastomosis due to the severe condition of the stricture. Moreover, the patient has been required to undergo feeding through a jejunum nutrient catheter since 2008.

### 2.2 Evaluation methods

#### 2.2.1 Clinical evaluation

The clinical evaluation has 2 components: The clinical information collection, which included the feeding method, length of feeding time per meal, the condition of choke/reflux, and the integrity of the medical history, each completed by a doctor; The clinical swallow examination, which including orofacial function, laryngeal and pulmonary function, gag reflex and response, and a trial swallowing function, each completed by a speech-language pathologist.

#### 2.2.2 Functional oral intake scale (FOIS)

The following scores (levels 1–7) describe the FOIS:

Level 1: No oral intake

Level 2: Tube dependent with minimal/inconsistent oral intake

Level 3: Tube supplements with consistent oral intake

Level 4: Total oral intake of a single consistency

Level 5: Total oral intake of multiple consistencies requiring special preparation

Level 6: Total oral intake with no special preparation, but must avoid specific foods or liquid items

Level 7: Total oral intake with no restrictions

#### 2.2.3 Videofluoroscopic swallowing study (VFSS), the digital acquisition, and analysis system of videofluoroscopy

The VFSS involves the use of a fluoroscopic X-ray image along with various consistencies of barium sulfate to evaluate the physiology of the swallowing function during the oral, pharyngeal, and esophageal phases. To examine the swallowing function of the patient in the seated position, the X-ray posteroanterior and lateral views were performed by fluoroscopy. The examination has 4 major components: accumulating oropharyngeal residual bolus levels; observing laryngeal penetration and tracheal aspiration; observing upper esophageal sphincter (UES) motility; and observing oral and nasal reflux.

The digital acquisition and analysis system of the videofluoroscopy is a type of method used to quantitatively analyze the VFSS video. By applying the video that was recorded during the VFSS and playing the video frame by frame, while observing the target organ (hyoid, larynx, UES), capturing the target frame (when the hyoid located at the lowest/highest point and the UES is maximally distended during swallowing), and measuring and calculating the illustrations using Image J software (National Institute of Mental Health, Bethesda, MD), the medical personnel can analyze the temporal and kinetic parameters to depict the swallowing ability of the patient in a more detailed manner. In this case, the parameters include the anterior hyoid excursion, superior hyoid excursion, UES opening, and pharynx constriction rate.^[7,8]^

#### 2.2.4 High resolution manometry

A solid-state manometric assembly with 36 circumferential sensors was used (Sierra Scientific Instruments, Los Angeles, CA) to collect the pressure from the oropharynx to the upper esophagus during swallowing at rest.

Pressure and timing data were initially analyzed using ManoView ESO 3.0 analysis software (Sierra Scientific Instruments). The pressure and temporal parameters examined included the pharyngeal maximum pressure (mm Hg), the duration of the pharyngeal pressure above baseline (millisecond), the duration of UES relaxation (millisecond), and the UES residual pressure (mm Hg).^[8]^

In this case, the UES of the patient was absent due to prior surgical procedures. However, the presence of the anastomotic stricture replaced the role of the UES.

### 2.3 Comprehensive swallowing function exercises for the patient

#### 2.3.1 Exercises to enhance the protective ability of airway

In this training, assistive devices were utilized to enhance airway protection. The TRI-BALL-breathing exerciser device was used twice a day. The patient underwent exhalation and inhalation 50 times each. The Sounder training was utilized twice a day in which vocal sounds were exerted 20 times each. The next step included the Maneuver treatment, which was the super-supraglottic swallow exercise. Following the Maneuver treatment, the patient underwent training of the vocal cord motion. To do this, the palms of the hand were tightly pressed together while simultaneously exerting vocal sounds.

#### 2.3.2 Exercises to strengthen the muscles associated with swallowing

In this training procedure, assistive devices were used to implement resistive training of the tongue muscle. Next, the Maneuver treatment was utilized, which consisted of the Masako Maneuver and an Effortful Swallow exercise. The 3rd portion of the muscle strengthening training exercise consisted of the VitalStim therapy system, which serves to stimulate the electrodes located on the suprahyoid and infrahyoid muscles twice a day for 20 minutes, 5 days a week.

#### 2.3.3 Swallowing maneuver modification

The modifications in swallowing involved in this training technique consisted of a deep inhalation that was tightly held, consumption of a small amount of food, lowering of the head, and intense swallowing while the head of the patient was lowered and breathing was withheld. This procedure was continued by the patient until swallowing was thoroughly completed. The exercise was conducted 10 times per week for 90 minutes each session, and the amount of food consumed was recorded for each meal.

#### 2.3.4 Training to prevent reflux

The patient was prohibited from decubitus for 1 hour after feeding. In addition, the patient was administered an acid inhibitor and gastro-kinetic agent.

## 3 Results

### 3.1 Clinical evaluation

Prior to the treatment that was employed in this study, the patient was fed through a jejunum nutrient catheter for a time period of 30 to 60 minutes per meal. Choking, oral reflux, and pharyngeal reflux occasionally occurred after feeding. The orofacial, laryngeal and pulmonary function, and gag reflex of the patient were normal. However, there remained a mass of bolus in the oral cavity following repeated swallowing.

**Figure 1:**
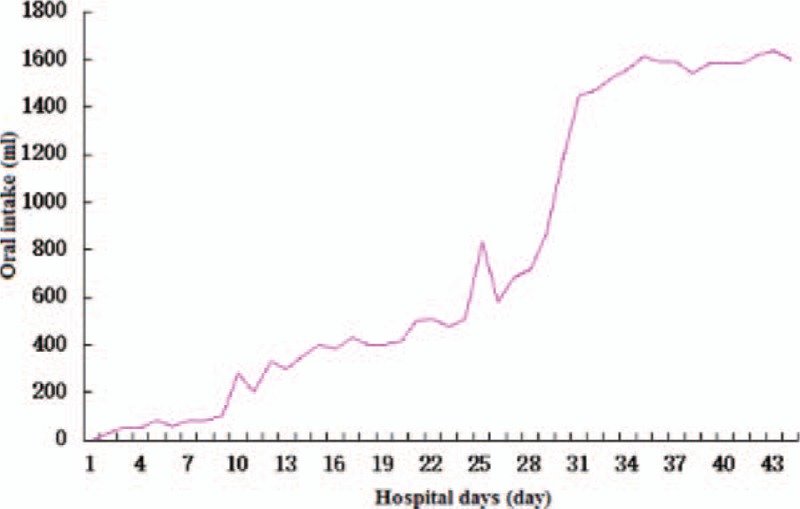
Daily oral intake during hospitalization.

The posttherapy outcomes of this study included the removal of the jejunum nutrient catheter, and the presentation of complete oral intake for a period of 3 to 4 minutes per meal (Fig. 1). There was no observation of choking, oral reflux, or pharyngeal reflux following the meal intake. Orofacial, laryngeal and pulmonary function, and gag reflex of the patient were normal. Furthermore, there was no indication of oral bolus residue following patient examination.

### 3.2 FOIS

Prior to the therapy employed in this study, the FOIS score of the patient was at level 1, which indicates no oral intake. Following therapy, the patient exhibited a FOIS score at level 7, which indicates total oral intake with no restrictions.

### 3.3 VFSS, the digital acquisition, and analysis system of videofluoroscopy

**Figure 2:**
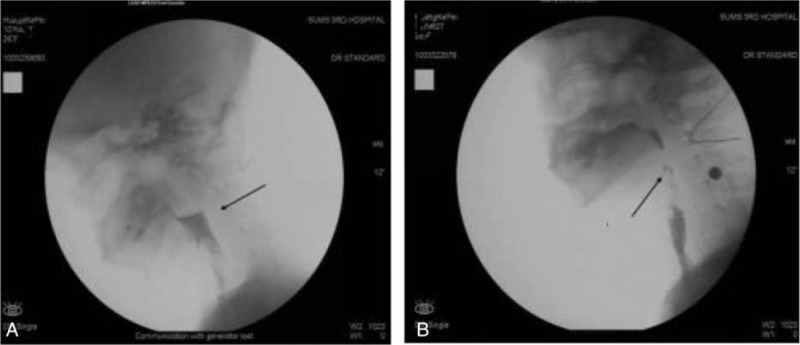
Residual bolus in the vallecula epiglottica and pyriform sinus. (A) Pretherapy, (B) posttherapy.

(1) VFSS: Prior to employing the training exercises to enhance the swallowing function of the patient, the patient exhibited a normal oral phase. In the pharyngeal phase, we observed a mass of residual bolus (Fig. 2A) in the vallecula epiglottica and pyriform sinus, as well as pooling above the anastomosis. In addition, laryngeal penetration, and an occasionally opened anastomosis was observed in the patient. The posttherapy VFSS results demonstrated a Normal oral phase. In the pharyngeal phase, the patient displayed a negligible amount of residual bolus (Fig. 2B) in the vallecula epiglottica. The patient did not display laryngeal penetration or tracheal aspiration. Furthermore, the extent to which the anastomosis opened was commensurate to average levels.

(2) Digital acquisition and system analysis of videofluoroscopy (Table 1)

**Table 1:**
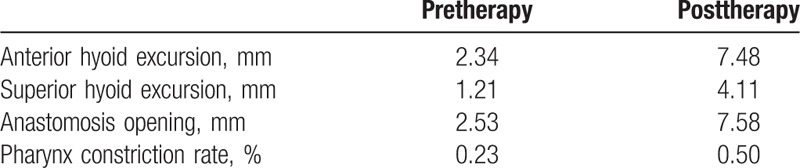
Results of digital acquisition and system analysis of videofluoroscopy.

### 3.4 High resolution manometry (Fig. 3 and Table 2)

**Figure 3:**
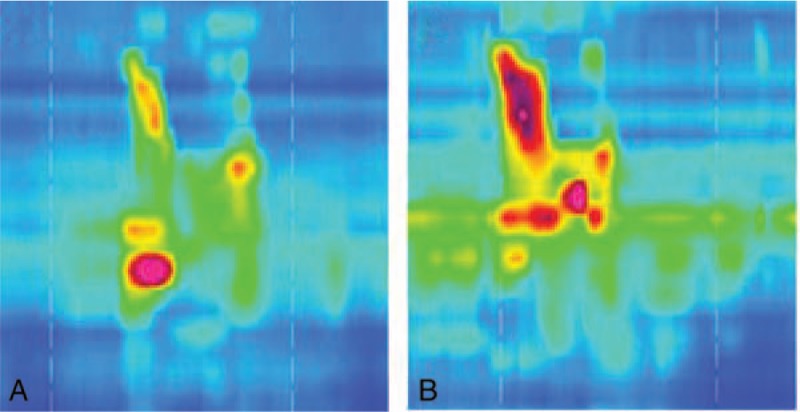
Space-time map of high resolution manometry. (A) Pretherapy, (B) posttherapy.

**Table 2:**
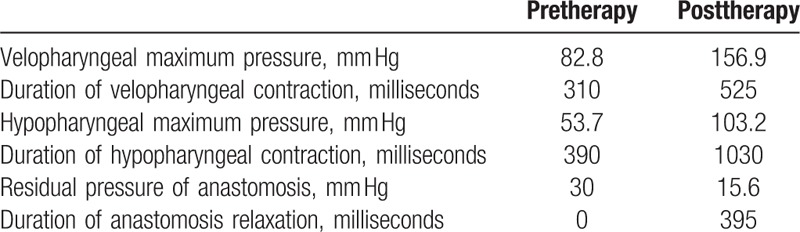
Results of high resolution manometry.

### 3.5 Nutrition state

The pretherapy weight of the patient was 47 kg, and the serum prealbumin level was 145 mg/L. Following therapy, the patient weighed 51 kg, and the serum prealbumin level was 174 mg/L.

## 4 Discussion

Under normal conditions in the physiological system, the UES is typically closed between oral swallows, and its opening is obligatory for swallowing to occur. The degree of UES relaxation has 3 major influential factors: oropharyngeal bolus propulsion (ie, intrabolus pressure), which is produced by the tongue base retraction and the pharyngeal constrictor contraction, propel bolus, and furthermore, stimulate the UES to relax; anterior hyolaryngeal traction, which is produced by the suprahyoid muscles contraction to tract the hyoid and tissue surrounding UES to move anteriorly and superiorly, mechanical stretches to open UES; and sphincter relaxation. Normally, the UES is relaxed and produce a negative pressure (ie, UES residual pressure) when the bolus get into pharyngeal. It is a positive factor to help bolus get into esophagus. However, it would become a high pressure zone and produce obstruction of the food pathway if the UES is failure to relaxation. Therefore, the UES opening is effected positively by oropharyngeal bolus propulsion and anterior hyolaryngeal traction.^[9]^ They should overcome obstruction from sphincterial achalasia so that the bolus can pass through the UES.^[10]^

In the present case, the patient underwent esophageal replacement. The UES was absent from the patient due to a previous operation. In addition, the narrow anastomotic stoma produced further mechanical resistance. The resistance played a similar role in which the bolus was hampered from entering the esophagus as with the UES.

Prior to the treatment conducted in this study, the VFSS demonstrated a mass of bolus residues in the vallecula epiglottica and the pyriform sinus, which indicates that the patient exhibited weak oropharyngeal bolus propulsion. The poor results obtained from the velopharyngeal/hypopharyngeal maximum pressure measurement and the anterior/superior hyoid excursion further provided evidence for a weakened bolus propulsion. The oropharyngeal bolus propulsion and the anterior hyolaryngeal traction were severely impaired and insufficient to compete with the resistance produced by the anastomotic stricture.

Previous studies indicated that esophagus dilatation is an effective method to improve dysphagia caused by esophageal stricture. However, some patients have to undergo recurrent treatments.^[11]^ In the present case, the patient suffered from dysphagia caused by anastomotic stricture. Furthermore, the results following recurrent esophageal dilation after the onset of dysphagia did not indicate an improved swallowing function.

On account of these occurrences, we formulated a plan of comprehensive swallowing function training to improve the oropharyngeal bolus propulsion and the anterior hyolaryngeal traction.

After 40 days of therapy, the VFSS demonstrated that a small amount of residual bolus was found in the vallecula epiglottica and pyriform sinus. Moreover, the extent to which the anastomosis opened was average. The nutritional needs of the patient were met daily through oral food intake. Furthermore, the jejunum nutrient catheter that was present in the patient prior to treatment was removed and the patient was able to advance to complete oral feeding. The transition of the patient from having no oral intake to complete oral feeding signifies vast improvement and further validates the utility of comprehensive swallowing function exercises.

## 5 Conclusion

The oropharyngeal bolus propulsion, anterior hyolaryngeal traction, and sphincter relaxation are 3 major factors needed to open the UES. However, in the present study, we examined a patient who suffered from anastomotic stricture. The impairment of the UES as well as the stricture is a synonymous feature that contributes to the hindrance of sufficient food intake, and further results in dysphagia. The enhancement of the oropharyngeal bolus propulsion, anterior hyolaryngeal traction to overcome resistance may serve as an effective approach aimed at improving the function of swallowing in the event that recurrent UES in combination with esophageal dilatation, is unsuccessful. The patient has succeeded with this method. To investigate the new treatment strategy for complicated dysphagia caused by esophageal replacement with colon, the further investigations are needed.
